# Deletion of the thrombin cleavage domain of osteopontin mediates breast cancer cell adhesion, proteolytic activity, tumorgenicity, and metastasis

**DOI:** 10.1186/1471-2407-11-25

**Published:** 2011-01-19

**Authors:** Michel S Beausoleil, Erika B Schulze, David Goodale, Carl O Postenka, Alison L Allan

**Affiliations:** 1 Department of Anatomy & Cell Biology, Schulich School of Medicine and Dentistry, University of Western Ontario, (1151 Richmond Street), London Ontario, (N6A 3K7), Canada; 2 Department of Oncology, Schulich School of Medicine and Dentistry, (790 Commissioners Road East), London Ontario, (N6A 4L6), Canada; 3 London Regional Cancer Program, London Health Sciences Centre, (790 Commissioners Road East), London Ontario, (N6A 4L6), Canada; 4 Lawson Health Research Institute, (375 South Street), London Ontario, (N6A 4G5), Canada

## Abstract

**Background:**

Osteopontin (OPN) is a secreted phosphoprotein often overexpressed at high levels in the blood and primary tumors of breast cancer patients. OPN contains two integrin-binding sites and a thrombin cleavage domain located in close proximity to each other.

**Methods:**

To study the role of the thrombin cleavage site of OPN, MDA-MB-468 human breast cancer cells were stably transfected with either wildtype OPN (468-OPN), mutant OPN lacking the thrombin cleavage domain (468-ΔTC) or an empty vector (468-CON) and assessed for *in vitro *and *in vivo *functional differences in malignant/metastatic behavior.

**Results:**

All three cell lines were found to equivalently express thrombin, tissue factor, CD44, αvβ5 integrin and β1 integrin. Relative to 468-OPN and 468-CON cells, 468-ΔTC cells expressing OPN with a deleted thrombin cleavage domain demonstrated decreased cell adhesion (p < 0.001), decreased mRNA expression of MCAM, maspin and TRAIL (p < 0.01), and increased uPA expression and activity (p < 0.01) *in vitro*. Furthermore, injection of 468-ΔTC cells into the mammary fat pad of nude mice resulted in decreased primary tumor latency time (p < 0.01) and increased primary tumor growth and lymph node metastatic burden (p < 0.001) compared to 468-OPN and 468-CON cells.

**Conclusions:**

The results presented here suggest that expression of thrombin-uncleavable OPN imparts an early tumor formation advantage as well as a metastatic advantage for breast cancer cells, possibly due to increased proteolytic activity and decreased adhesion and apoptosis. Clarification of the mechanisms responsible for these observations and the translation of this knowledge into the clinic could ultimately provide new therapeutic opportunities for combating breast cancer.

## Background

Breast cancer is a leading cause of cancer death in women, mainly due to metastasis of the disease to distant organs. How the process of metastasis occurs, and what biological factors may contribute to it, is an important field of research being undertaken. It is believed that with improved understanding of the biology of the disease we can develop new diagnostic, prognostic and therapeutic methods. The current study is focused on examining the interaction of two such factors; thrombin and osteopontin (OPN).

OPN has been clinically associated with many types of human cancer [1-10]. Specifically in breast cancer patients with metastatic disease, elevated levels of baseline OPN in plasma have been linked to poor prognosis [11,12]. Other studies have found that changes in OPN plasma level over time after therapy are associated with clinical outcome [13]. Experimentally, OPN has been functionally associated with growth, survival, adhesion, migration, invasion, angiogenesis and metastasis of breast cancer cells [14-24]. Furthermore, OPN has been shown to interact with cell surface receptors (integrins, CD44) [15,19,21,25], secreted proteases (urokinase plasminogen activator, thrombin) [17,24] and growth factor/receptor pathways (EGFR, Met) [16,18] in order to exert its malignancy-promoting effects.

OPN has many protein interaction domains which are thought to play a role in the function of the protein. These include two integrin binding sites (RGD [arginine^159^- aspartic acid^161^] and SVVYLR [serine^162^-arginine^168^]; a CD44 binding domain; and a thrombin cleavage domain (RSK [arginine^168^-lysine^170^]) [26]. When OPN is cleaved at the RSK site by thrombin, it is separated into two approximately equivalent sized pieces, including N-terminal and C-terminal fragments [27]. Thrombin itself is a secreted serine protease found in the blood and an integral protein in the processes of haemostasis and coagulation [28]. Thrombin is activated upstream by tissue factor (TF) which is exposed on the surface of endothelial cells after injury, but is also often overexpressed on the surface of cancer cells [29]. The tumor microenvironment thus provides a rich environment for abundant activation of thrombin and therefore OPN cleavage.

The effect of OPN cleavage by thrombin has been previously studied by ourselves and others [24,27,30,31]. Senger et al. [27], demonstrated that when OPN is cleaved by thrombin, *in vitro *adhesion and migration of cancer cells is increased, specifically due to the N-terminal domain of OPN, possibly by increasing access to the integrin binding domains [27,30]. However, work by Mi et al. [31] observed that it is the C-terminal domain of thrombin-cleaved OPN that increases both migration and adhesion of breast cancer cells. This C-terminal effect occurs by complexing with cyclophilin C and binding of CD147 on the cell surface [31]. Our laboratory has previously shown that blockage of thrombin activity using Argatroban (a clinically used thrombin inhibitor) specifically reduced adhesion and migration of MDA-MB-468 breast cancer cells transfected with OPN (468-OPN) but had no effect on control cells (468-CON) [24]. Furthermore, Argatroban treatment of tumor-bearing mice reduced primary tumor growth, lymphovascular invasion, and lymph node metastasis using both OPN-dependent and OPN-independent mechanisms [24]. This data from our lab clearly shows that pharmacologic inhibition of thrombin activity can reduce OPN-mediated metastatic behavior. However, the specific biochemical interaction between thrombin and osteopontin and its role in mediating breast cancer cell malignancy requires further elucidation.

In the current study, we investigated the biochemical and functional relationship between OPN and thrombin during breast cancer metastasis using the 468-CON and 468-OPN human breast cancer cell lines, as well as a third cell line (468-ΔTC) which expresses a mutant OPN lacking the thrombin cleavage domain. In doing this, we hoped to identify those effects of OPN that were due to its direct thrombin cleavage rather than effects simply related to pharmacological inhibition of host- and/or tumor cell-produced thrombin. We hypothesized that deletion of the thrombin cleavage site on the OPN protein backbone would alter the ability of OPN to mediate breast cancer malignancy and metastasis. Unexpectedly, the novel findings presented here indicate that breast cancer cells expressing thrombin-uncleavable OPN demonstrate enhanced metastatic behavior *in vitro *and *in vivo *relative to cells expressing wildtype OPN.

## Methods

### Cell Culture

The tumorigenic, weakly metastatic MDA-MB-468 human breast cancer cell line (a kind gift from Dr. Janet Price, University of Texas, M.D. Anderson Cancer Center, Houston, TX) was maintained in αMEM medium (Invitrogen; Carlsbad, CA) supplemented with 10% fetal bovine serum (FBS, Sigma Chemical Company; St. Louis, MO) at 37°C in a humidified CO_2 _incubator.

### Plasmids & Transfection

Stable transfection of MDA-MB-468 human breast cancer cells was carried out using liposome-based transfection as described previously ([21]. Plasmids for transfections included an unmodified pcDNA3 control plasmid (Invitrogen); a wildtype OPN expression vector that was previously generated by cloning the full-length human OPN cDNA (from plasmid OP-10) into pcDNA3 [32,33]; and a mutant OPN expression vector in which the thrombin cleavage region (^166^GLRSKS^171^) was deleted. The thrombin-uncleavable OPN expression construct was generated by site-directed mutagenesis of the wildtype OPN expression plasmid using a 49 mer mutagenic oligonucleotide specific for introducing the thrombin cleavage deletion (ΔTC) (5' GGATGTCAGGTCTGCGAAACTTCTTATAAACCACACTATCACCT CGGCC 3'). In order to control for clonal heterogeneity, pooled populations were created by combining several clonal cell populations that resulted from the stable transfections. The specific cell populations generated included a control vector-transfected MDA-MB-468 pooled population consisting of 6 clones (468-CON), an OPN-transfected MDA-MB-468 pooled population consisting of 6 clones (468-OPN) [21] and a ΔTC-OPN-transfected MDA-MB-468 pooled population consisting of 6 clones (468-ΔTC). To maintain stable transgene expression, cell lines were maintained under selective pressure using 500 μg/ml of active Geneticin (G418-sulfate, Invitrogen).

### Western Blot Analysis

Cells were plated at 10^6 ^cells/100 mm plate, grown for 48 hrs and media was changed to Opti-MEM media (Invitrogen). Conditioned media was collected after 18 hrs and cells were counted. For each cell line, a normalized volume of conditioned media equivalent to 5 × 10^5 ^cells was subjected to electrophoresis in 10% SDS-polyacrylamide gels and transferred onto polyvinylidene difluoride membranes (Immobilon™, Millipore; Bedford, MA). After transfer, gels were stained with Coomassie Blue to confirm equal loading and transfer efficiency. Primary antibodies included a sheep polyclonal anti-human antibody against tissue factor (TF, Cedarlane Laboratories; Burlington, ON, 1:2000 dilution), a mouse monoclonal anti-human antibody against thrombin (clone 12, BD Biosciences; Franklin Lakes, NJ, 1:1000 dilution), and a rabbit monospecific polyclonal anti-human antibody against OPN (hOPN1; 7.5 μg/ml), a kind gift from Dr. Toshi Uede, Hokkaido University, Sapporo, Japan [34]. The secondary antibodies used were anti-sheep (TF), anti-mouse (thrombin) (Sigma) or anti-rabbit (OPN) (GE Healthcare, Little Chalfont Buckinghamshire, UK) HRP conjugates (1:2000 dilution). The blocking/dilution reagent used was 5% skim milk in TBST (Tris-buffered saline + 0.05% Tween-20). Thrombin, TF, and OPN protein expression was visualized using an enhanced chemiluminescence system (Roche Applied Sciences; Laval, QC).

### Flow Cytometry Analysis

Cells were grown to ~80% confluence in normal media and harvested by gentle trypsinization. Flow cytometry analysis of cell surface receptors was carried out using the appropriate primary antibodies against integrins: β_1_, β_3_, α_v_β_5_, α_9_β_1_, (Chemicon) or IgG (negative isotype control) (1 μg/10^6 ^cells) (Cedarlane, Mississauga, ON) for 1 hr at 4°C and phycoerytherin (PE)-labeled secondary antibody (BD Biosciences, Franklin Lakes, NJ) for 1 hr in the dark. Samples were run on a Beckman Coulter EPICS XL-MCL flow cytometer. For CD44 expression analysis, CD44-PE and IgG-PE (negative isotype control) (BD Biosciences) antibodies were used and analyzed in a similar fashion.

### Cell Adhesion Assay

Cells were plated onto sterile 96-well non-tissue culture plates (Titertek, Flow Laboratories Inc.; McLean, VA) coated with either 5 μg/ml of human vitronectin (VN; Sigma) or PBS (negative control), using 1 × 10^4 ^cells/well in triplicate wells for each treatment. The experiment was completed three times. Vitronectin was chosen because it is a ligand for αvβ1 and αvβ5, two integrins that are known to interact with OPN (via the RGD region), and that are expressed by MDA-MB-468 cells. For each cell line, experiments were conducted in the presence or absence of 25 μg/ml Argatroban (pre-treatment period of 15 min) or 25 μg/ml integrin blocking antibodies (α_v_β_5 _or β_1_) (Chemicon, Temecula, CA). Cells were allowed to adhere for 5 h at 37°C. Adhered cells were then fixed using fresh 2% gluteraldehyde/PBS and stained using Harris' hematoxylin. Blue stained cells were manually counted using a light microscope. Five high-powered fields (HPF) of view were counted for each well, and the mean number of cells per HPF were calculated and compared. Argatroban (Abbott Laboratories; North Chicago, IL) was purchased through the London Health Science Centre pharmacy.

### Cell Growth Assays

Cells were plated at a density of 5.0 × 10^4 ^cells/60 mm plate (n = 3 for each time point) and maintained in regular growth media. Every 48 h for 12 days, triplicate cultures were trypsinized and manually counted for viable cells using a hemocytometer and trypan blue exclusion.

### Cell Migration Assays

In preparation for the assay, 6.5 mm Transwell plates with a 8.0 μm pore size (Becton Dickinson; Franklin Lakes, NJ) were pre-coated with gelatin (6 μg/well, n = 3 wells per experimental group). Media containing a chemoattractant (10% FBS) or serum-free media + 0.1% BSA (negative control) was added to the bottom chamber of the Transwell plates. Cell suspensions (5.0 × 10^4 ^cells/well in serum-free growth medium + 0.1% BSA) were added to the top of the wells and allowed to incubate at 37°C for 24 h. The upper portion of the transwell was removed, inverted, and fixed using fresh 1% gluteraldehyde/PBS and stained using Harris' hematoxylin. Wells were rinsed with distilled water and any non-migrated cells were carefully removed with a cotton swab. Five HPF of view were manually counted for each well, and the mean number of cells per HPF was calculated and compared.

### Semi-Quantitative Zymography

Conditioned media were prepared as described in the western blot methodology. For each cell line, a normalized volume of conditioned media equivalent to 5 × 10^3 ^cells was subjected to electrophoresis in 10% SDS-polyacrylamide gels supplemented with 2 mg/ml of casein and 5 μg/ml of plasminogen (Sigma). Gels were washed twice for 15 min each in 2.5% Triton X-100 solution to remove SDS followed by 5 washes in water. Enzymatic activity was measured after 18 hr of incubation at 37°C in incubation buffer (50 mM Tris-HCl and 5 mM CaCl_2_, pH = 7.0). Finally, gels were stained with Coomassie Blue for 1 hour and destained. White bands representing enzymatic activity were measured by densitometric analysis using a BioRad Universal Hood II and Quantity One 4.6.1 software (BioRad). Density measurements were normalized to 468-CON samples for each biological replicate and pooled for analysis.

### *In Vivo *Tumorigenicity and Metastasis Assays

Animal research conformed to the Helsinki Declaration and to local legislation. All animal procedures were conducted in accordance with the recommendations of the Canadian Council on Animal Care, under a protocol approved by the University of Western Ontario Council on Animal Care.

Cells were prepared in sterile PBS at a concentration of 1 × 10^6 ^cells/100 μl and injected into the second thoracic mammary fat pad of 7-8 week-old female athymic nude (*nu/nu*) mice as described elsewhere, using 1 × 10^6 ^cells/mouse and 12 mice/treatment group [21]. Primary tumor growth was evaluated weekly by caliper measurement in two perpendicular dimensions, and tumor volume was estimated using the following formula: [volume = 0.52 × (width)^2 ^× (length)] for approximating the volume (mm^3^) of an ellipsoid. Differences in mean tumor volume were analyzed for each weekly time point.

As per previously established endpoints [21], mice were sacrificed at approximately 12 weeks post-injection and assessed for metastatic burden. Tissues and organs were examined superficially for evidence of gross macroscopic metastases at necropsy, prior to processing for histology. Primary tumors and mouse tissues collected at necropsy were fixed in 10% neutral-buffered formalin before processing. Tissues were embedded in paraffin wax, sectioned (4 μm thick), and subjected to standard hematoxylin and eosin (H&E) staining. Stained slides were evaluated by light microscopy in a blinded fashion by an experienced pathologist in order to observe histopathological characteristics and to identify regions of micrometastatic involvement. Color RGB (Red Green Blue) images were taken using an Olympus^© ^microscope (Olympus America, Center Valley, PA) and regions of micrometastatic involvement were manually outlined. Metastatic tumor burden in the lymph node (tumor area/organ total area) was determined quantitatively using ImageJ analysis software (NIH). Color images were converted to 8-bit images and thresholding was performed on each picture to obtain a black (manually outlined total organ tissue or total tumor tissue) and white (background) image. Quantification was done using the 'analyze particles' option of the ImageJ software after the scale had been set for the microscope objective used.

### RNA Extraction and Quantitative Real-Time PCR

Cells were grown on 60 mm diameter dishes until ~80% confluence (3 dishes/cell line) and total RNA was collected using the TRIzol^® ^Reagent (Invitrogen) system as per the manufacturer's protocol. Total RNA was quantified by spectrophotometry, assuming an absorbance (at 260 nm) of 1.0 equal to 40 μg RNA/mL. cDNA was created from 1 μg of total RNA using SuperScript III Reverse transcriptase, oligo(dt)20 primers, 10 mM dNTP Mix, 5× Strand Buffer, 0.1 M DTT and RNase out (Invitrogen). Reverse transcription (no SuperScript III) were run with every reaction group.

Real-time PCR was conducted with a Rotor-Gene 3000 temperature cycler (Corbett Research, San Francisco, CA) using cDNA as a template and Brilliant^® ^SYBR^® ^Green QPCR Master Mix (Stratagene), following the manufacturer's protocol. Expression was normalized to glyceraldehyde 3-phosphate dehydrogenase (GAPDH) internal control and further compared to the 468-CON cell line. Reverse transcription (no SuperScript III) and non template (no cDNA) controls were run with every reaction to ensure that genomic DNA was not amplified and the reagents were not contaminated. Primers used: GAPDH [Forward 5'-CATGTTCGT CATGGGTGTGAACCA-3', Reverse 5'-ATGGCATGGACTGTGGTCATGAGT-3']; uPA [Forward 5'-CAGGGCATCTCCTGTGCATG-3', Reverse 5'-AGCCCTG CCCTGAAGTCGTTA-3']; MCAM [Forward 5'-GGGTACCCCATTCCTCAAGT-3', Reverse 5'-CCTGGACTCCTTCATGTGGT-3']; Maspin (SERPINB5) [Forward 5'-CTACTTTGTTGGCAAGTGGATGAA-3', Reverse ACTGGTTTGGTGTCTGTC TTGTTG-3']; and TRAIL (TNFSF10) [Forward 5'-CGTGTACTTTACCAACGAG CTGA-3', Reverse 5'-ACGGAGTTGCCACTTGACTTG-3'].

### Statistical Analysis

All *in vitro *experiments were performed at least in triplicate, and data were compiled from 3 separate experiments. *In vivo *studies were carried out using multiple animals (n = 12 per experimental group). Statistical analysis was performed using GraphPad Prism 4.0 software^© ^(San Diego, CA) using ANOVA with Tukey post hoc test (for comparison between more than two groups). Growth curve analysis was done using Prism 4.0, using non-linear regression with an F-test. In all cases, values of p ≤ 0.05 were regarded as being statistically significant.

## Results

### Characterization of OPN, thrombin, TF, and OPN-related receptor expression in transfected MDA-MB-468 cells

**Figure 1:**
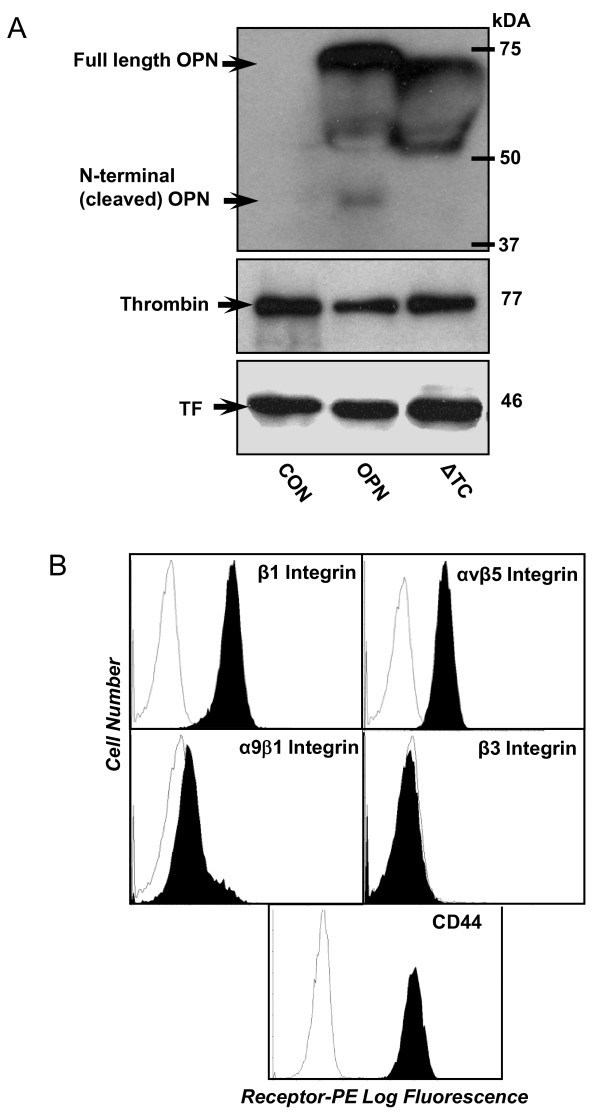
***In vitro *protein expression of OPN, thrombin, TF, and OPN-related receptors in 468-CON, 468-OPN and 468-ΔTC cells**. ***(A) ***Protein expression of OPN, thrombin, and tissue factor (TF) was analyzed by western blot analysis of conditioned media from 5 × 10^5 ^cultured cells for each cell line as described in the Materials and Methods. ***(B) ***Receptor expression was measured by flow cytometry analysis. Cultured cells were incubated with specific antibodies (*filled profiles*) or with a nonspecific isotype control primary antibody (*open profiles*). Filled profiles represent expression of α9β1 integrin, β1 integrin, β3 integrin, αvβ5 integrin and CD44. Representative results for 468-CON cells only are shown; equivalent expression was observed in all cell lines (*data not shown*).

MDA-MB-468 breast cancer cells were stably transfected to overexpress either wildtype OPN (468-OPN), thrombin-uncleavable OPN (468-ΔTC) or an empty vector (468-CON) [21]. The resulting cell lines were characterized for their expression of the secreted proteins OPN, thrombin, and TF using western blot analysis (Figure 1A) and for the OPN-related receptors β1 integrin, β3 integrin, αvβ5 integrin, α9β1 integrin and CD44 using flow cytometry analysis (Figure 1B).

All three cell lines strongly expressed both thrombin and TF at approximately equivalent levels, indicating that they would be present in the *in vitro *tissue culture environment during experimental assays (Figure 1A). Relative to 468-CON cells, 468-OPN and 468-ΔTC cells expressed high levels of OPN protein (Figure 1A). However, only the 468-OPN cells expressed the N-terminal cleavage product of OPN, indicating that the thrombin expressed by the cells is capable of cleaving only the wildtype OPN produced by 468-OPN cells and not the thrombin uncleavable OPN produced by 468-ΔTC cells. In addition, the OPN expressed by the 468-ΔTC cells ran slightly smaller than the wildtype indicating its smaller size due to the deleted region (Figure 1A). Interestingly, we observed an additional ~55 kDa band expressed by 468-ΔTC cells, which may represent a novel post-translational modification.

It was also found that each cell line equivalently expressed CD44, αvβ5 integrin and β1 integrin, but not β3 or α9β1 integrins. (Figure 1B). Representative results for 468-CON cells only are shown; however equivalent expression was observed in all cell lines (*Additional File *1, *Figure S1*). Therefore any differences seen between the cell lines in subsequent experimental assays would not be due to variable receptor expression.

### Deletion of the thrombin-cleavage region of OPN reduces *in vitro *cell adhesion of MDA-MB-468 cells

**Figure 2:**
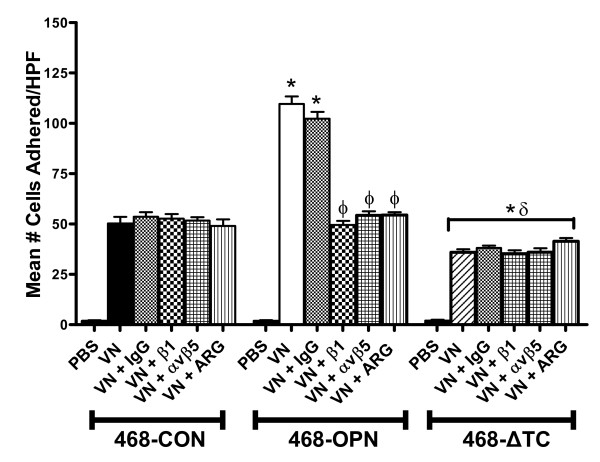
***In vitro *cell adhesion of 468-CON, 468-OPN and 468-ΔTC cells**. 96 well plates were pre-coated with vitronectin (5 μg/ml) or PBS (neg. control). 1 × 10^4 ^cells/well and 3 wells/treatment were plated and allowed to adhere for 5 hrs. Experiments were carried out in the presence or absence of Argatroban (25 μg/ml), or blocking antibodies against integrins β1 or αvβ5 or IgG (25 μg/ml). Adhered cells were quantified by manual counting of 5 high powered fields. Data is presented as the mean +/-SEM. * = sig. different than corresponding treatment of 468-CON (p < 0.001); δ = sig. different than corresponding treatment of 468-OPN (p < 0.001); Φ = sig. different than respective vehicle or control (p < 0.05).

*In vitro *assays were carried out to measure adhesion of the cell lines to vitronectin (VN), a ligand for αvβ5 and αvβ1 integrins expressed by the MDA-MB-468 cells. Treatments consisted of Argatroban (thrombin inhibitor, 25 μg/ml), β1 or αvβ5 integrin blocking antibodies, or IgG (negative control) (25 μg/ml). Interestingly, the 468-OPN cells exhibited significantly increased adhesion compared to 468-CON and 468-ΔTC cells (p < 0.001), whereas the 468-ΔTC cells demonstrated significantly decreased adhesion compared to both 468-CON and 468-OPN cells (p < 0.001) (Figure 2). The increased adhesion in 468-OPN cells was dependant on thrombin cleavage as evidenced by the significant decrease of these behaviors when treated with the thrombin inhibitor Argatroban (p < 0.05). Treatment with β1 or αvβ5 blocking antibodies also significantly decreased adhesion of 468-OPN cells (p < 0.05), whereas the IgG isotype control antibody had no effect. Argatroban and integrin blocking antibodies had no effect on 468-ΔTC cell adhesion.

No difference was observed between 468-CON, 468-OPN, and 468-ΔTC cell lines with regards to cell proliferation *in vitro *(*Additional File *2, *Figure S2A*). Although we did observe that both 468-OPN and 468-ΔTC were significantly more migratory relative to 468-CON cells, there was no significant difference in migration between 468-OPN and 468-ΔTC cells (*Additional File *2, Figure S2B). This data is consistent with our previous studies using the 468-CON and 468-OPN cell lines [21,24].

### Deletion of the thrombin-cleavage region of OPN enhances *in vitro *proteolytic activity of MDA-MB-468 cells

**Figure 3:**
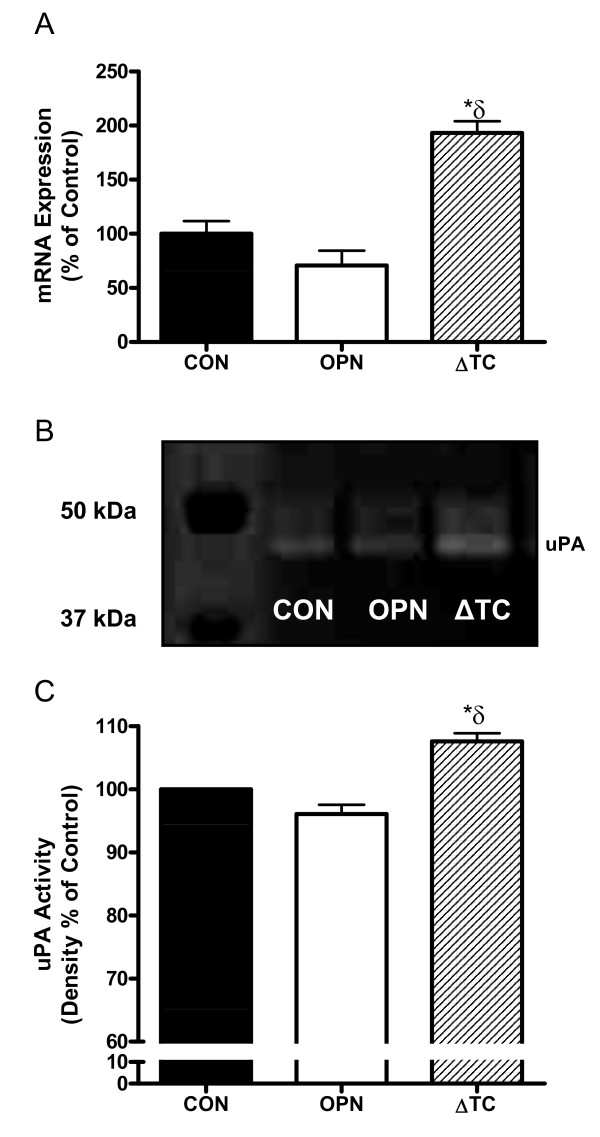
**uPA expression and proteolytic activity of 468-CON, 468-OPN, and 468-ΔTC cells**. ***(A) ***Quantitative RT-PCR of uPA mRNA levels collected from cells grown on 3 separate 60 mm dishes over 3 experiments. Expression was adjusted to GAPDH internal control. ***(B) ***Representative gel of zymographic analysis. ***(C) ***Conditioned media equivalent to 5 × 10^3 ^cells was subjected to zymographic analysis and density of bands was measured using BioRad Universal Hood II and Quantity One 4.6.1 software (BioRad). Experiments were replicated 3 times. Data represents % control of 468-CON cells and is presented as the mean +/- SEM. * = sig. different than corresponding treatment of 468-CON (p < 0.01); δ = sig. different than corresponding treatment of 468-OPN (p < 0.001).

To determine any proteolytic differences between the transfected cell lines, urokinase plasminogen activator (uPA) expression and activity were measured using quantitative real-time PCR and semi-quantitative zymography with subsequent densitometric analysis. The 468-ΔTC cells displayed significantly increased uPA expression (Figure 3A) and activity (Figure 3B,C) relative to both the 468-CON (p < 0.01) and 468-OPN (p < 0.001) cells. No differences in uPA expression or activity were observed between the 468-CON and 468-OPN cells.

### Deletion of the thrombin-cleavage region of OPN decreases tumor latency, increase primary tumor growth, and enhances lymph node metastasis of MDA-MB-468 cells *in vivo*

**Figure 4:**
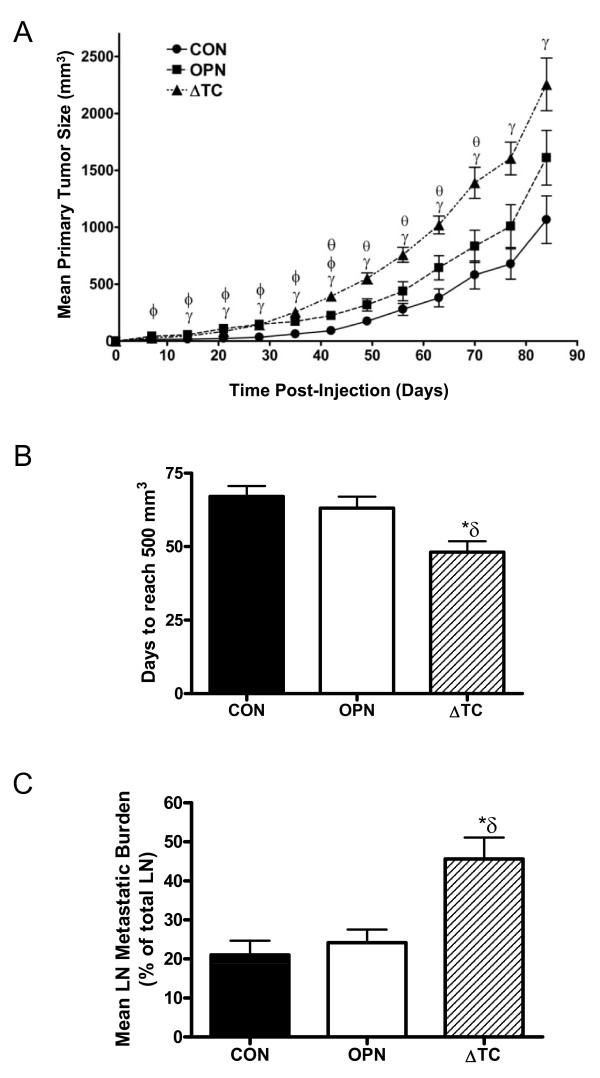
***In vivo *primary tumor growth and lymph node metastasis of 468-CON, 468-OPN and 468-ΔTC cells**. ***(A) ***Transfected cell lines were injected into the mammary fat pad of female nude mice using 1 × 10^6 ^cells/mouse and 12 mice/group. Tumors were measured weekly in two dimensions for twelve weeks using standard calipers and the tumor volume was estimated using the formula: [volume = 0.52 × (width)^2 ^× (length)]. Growth data are presented as mean +/- SEM. ***(B) ***Average tumor latency (time to reach 500 mm^3^) is represented as mean +/- SEM. ***(C) ***Mean lymph node metastatic burden (% of lymph node occupied by tumor) +/- SEM. At twelve weeks post-injection mice were sacrificed and assessed for lymph node metastasis. Tissue sections from primary lymph nodes (LN) were subjected to H&E staining and read by an experienced pathologist in a blinded fashion. γ = 468-ΔTC significantly different from 468-CON (p < 0.01), Φ = 468-OPN significantly different from 468-CON (p < 0.05), θ = 468-ΔTC significantly different from 468-OPN (p < 0.05), * = sig. different than corresponding treatment of 468-CON (p < 0.01); δ = sig. different than corresponding treatment of 468-OPN (p < 0.05).

To assess how OPN and thrombin-uncleavable OPN affect initiation and growth of primary tumors and metastases, an *in vivo *mouse model consisting of athymic nude (*nu/nu*) mice was used. Twelve mice per transfected cell line were injected with 1 × 10^6 ^cells into the mammary fat pad and tumor volume was measured weekly. With the exception of one mouse in the 468-CON group, all mice developed tumors. Initially, mice injected with both 468-OPN (p < 0.05) and 468-ΔTC (p < 0.001) cells grew significantly larger tumors than mice injected with 468-CON cells (Figure 4A). However, after day 42 the significant difference in tumor size between the 468-OPN and 468-CON groups was lost. At day 42, the 468-ΔTC group began growing tumors significantly larger than the 468-OPN and 468-CON groups (p < 0.05) (Figure 4A). The final mean tumor volume of the 468-ΔTC group was 2255.4 +/- 231.1 mm^3^; significantly larger than the 468-CON group, whose final mean tumor volume was 1066.2 mm^3 ^+/- 209.5 (p < 0.01).

Interestingly, the mean tumor latency time (time to reach 500 mm^3^) was significantly shorter for the 468-ΔTC group compared to both the 468-CON (p < 0.01) and 468-OPN groups (p < 0.05) (Figure 4B). However, the rate of tumor growth (i.e. doubling time) for each group was not observed to be significantly different when analyzed using non-linear regression analysis (*data not shown*).

At twelve weeks post-injection mice were sacrificed and assessed for metastatic characteristics. Tissue sections from lymph nodes (LN) were subjected to H&E staining and read by an experienced pathologist in a blinded fashion. The severity of metastasis in those lymph nodes which had been deemed positive for tumor cells was determined by measuring tumor burden. Specifically, mean LN metastatic burden (% of LN occupied by tumor) was calculated and showed a significantly higher burden in mice injected with 468-ΔTC cells than those injected with either 468-CON or 468-OPN cells (p < 0.001) (Figure 4C).

### Differential expression of MCAM, Maspin and TRAIL is consistent with the functional differences observed between 468-CON, 468-OPN, and 468-ΔTC cells

**Figure 5:**
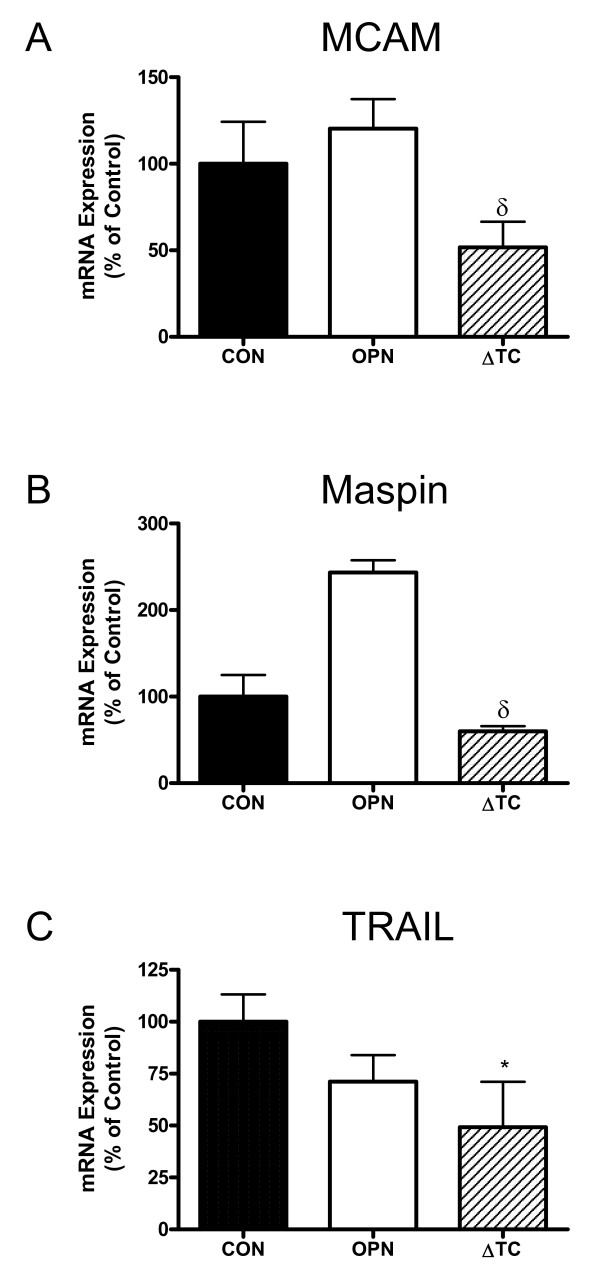
**mRNA expression of MCAM, Maspin and TRAIL in 468-CON, 468-OPN and 468-ΔTC cells**. Quantitative RT-PCR analysis of mRNA levels collected from cells grown on 3 separate 60 mm dishes over 3 experiments. Expression was adjusted to GAPDH internal control. ***(A) ***MCAM, ***(B) ***Maspin, and ***(C) ***TRAIL expression are represented as % control of 468-CON, mean +/- SEM. * = sig. different than corresponding treatment of 468-CON (p < 0.01); δ = sig. different than corresponding treatment of 468-OPN (p < 0.01).

Finally, to identify genes potentially involved in the functional differences observed between the transfected cell lines, a preliminary gene expression analysis was undertaken using a Human Genome U133 Plus 2.0 Array (*data not shown*). Analysis is ongoing, however several potential genes were identified that may play a mechanistic role in the effects observed in 468-ΔTC cells and were subsequently validated using quantitative real-time PCR (Figure 5). Expression of the adhesion molecule MCAM was found to be significantly decreased in 468-ΔTC cells compared to 468-OPN cells (p < 0.01) (Figure 5A). This is consistent with the functional effects observed, whereby 468-ΔTC cells showed significantly less cell adhesion than 468-OPN cells (Figure 2). The same was true of the uPA inhibitor maspin (SERPINB5), which was significantly decreased in 468-ΔTC cells compared to 468-OPN cells (p < 0.001) (Figure 5B). Again, this is consistent with the increased uPA expression and proteolytic activity in the 468-ΔTC cells observed *in vitro *(Figure 3) and the increased metastasis observed *in vivo *(Figure 4C). Lastly, the pro-apoptotic protein TNF-related apoptosis inducing ligand (TRAIL) which is encoded by the gene TNFSF10 was significantly reduced in 468-ΔTC compared to control (p < 0.01) (Figure 5C), consistent with the decreased tumor latency and increased tumor size observed in the *in vivo *studies (Figure 4A,B).

## Discussion

The secreted phosphoprotein OPN has been shown to be both prognostically and functionally important for breast cancer progression and metastasis [11-24]. However, the mechanistic ways in which OPN exerts these effects on breast cancer cells is still not well understood. Previous studies from our lab using the direct thrombin inhibitor Argatroban suggested that thrombin activity and subsequent cleavage of OPN is important for OPN to promote cancer progression and metastasis [24]. These results were focused more on globally knocking down host and/or tumor cells thrombin activity and indirectly observing the effect on OPN cleavage. In contrast, the current study focused specifically on OPN cleavage by comparing stably transfected cell lines expressing either wildtype OPN or a mutant OPN lacking the thrombin cleavage domain. Our novel findings indicate that loss of the thrombin cleavage domain actually promotes breast cancer cell progression and metastasis both *in vitro *and *in vivo *to a level exceeding that of wildtype OPN.

Using the stably transfected cell lines 468-OPN, 468-ΔTC and 468-CON, we investigated how thrombin cleavage of OPN impacts breast cancer cell function using *in vitro *and *in vivo *experiments representative of behaviors important to breast cancer progression and metastasis. Each cell line was characterized for expression of various OPN-related proteins. All three cell lines equivalently expressed thrombin, TF, CD44, αvβ5 and β1 integrins, therefore ensuring any differences in behavior observed were due to differences in OPN and not to differences in OPN-related proteins. It was also confirmed that the 468-OPN cell line expressed both full length and cleaved OPN, whereas the 468-ΔTC cells express only the full length protein. Interestingly, we observed an additional ~55 kDa band expressed by 468-ΔTC cells. Due to post-translational modifications such as glycosylation/sialation and phosphorylation, the molecular weight of OPN in monomeric form varies widely (41-75 kDa) [22]. However, the numerous forms of post-translationally modified OPN are very poorly understood, particularly with regards to their differential functional effects on cell behavior. It is possible that the additional ~55 kDa band expressed by 468-ΔTC cells represents a novel post-translational modification, and that this is related to the observed downstream functional effects.

*In vitro *analysis comparing the adhesive capabilities of the cells indicated that wildtype OPN increases adhesion of breast cancer cells whereas ΔTC-OPN decreases adhesion. The increased adhesion caused by wildtype OPN was specific to thrombin activity and integrin binding. This is in keeping with previous studies showing that pre-cleaved N-terminal OPN (containing the integrin binding sites) demonstrates increased adhesion over full-length wildtype OPN [24,27,30]. In contrast, the 468-ΔTC cells had decreased adhesive capabilities independent of thrombin or integrin blocking. Interestingly, the expression of the adhesion molecule MCAM by 468-ΔTC cells was also decreased significantly compared to 468-OPN cells. Loss of MCAM expression has been hypothesized to increase detachment of cells and therefore increase metastatic ability [35].

Urokinase plasminogen activator (uPA) is an important mediator of matrix degradation and invasiveness of cancer cells [36]. Both expression and activity of uPA was significantly elevated in 468-ΔTC cells. Also, expression of the uPA inhibitor and tumor suppressor maspin [37] was significantly higher in 468-OPN cells compared to 468-ΔTC cells, consistent with the increased uPA expression and proteolytic activity in 468-ΔTC cells observed *in vitro *and the increased metastasis observed *in vivo*. This suggests that ΔTC-OPN is capable of modulating several components of the uPA system. Wildtype OPN has previously been shown to increase uPA activity; however those studies used shorter treatments of exogenous OPN from various sources such as bacteria and human milk [17,32,38,39] rather than endogenously expressed OPN. There is still uncertainty in the literature concerning the post-translational modifications of OPN, and specifically the differences in host versus tumor derived OPN [40], which may also account for differences in observed results.

Although *in vitro *functional assays help us to describe individual behaviors of cancer cells, the human body is a complex system. We therefore also compared the cell lines using *in vivo *mammary fat pad injections and spontaneous metastasis assays in nude mice. Previous studies have demonstrated that there is a role for OPN in *in vivo *primary tumor growth of multiple cancer types [41-46]. Specifically in breast cancer, there are numerous reports of OPN knockdown that lead to decreased *in vivo *tumor growth [47-49]. Previous studies from our group and others aimed at blocking OPN cleavage *in vivo *by pharmacologically inhibiting thrombin indicate that inhibition of thrombin can reduce malignant and metastatic behavior of breast cancer cells using both OPN-dependent and OPN-independent mechanisms [24,50]. The current study found that directly removing the thrombin cleavage domain from OPN produced significantly larger tumors in a shorter time span than wildtype OPN or control. Also, the growth rates (i.e. doubling time) were not significantly different, indicating that thrombin-uncleavable OPN may grant breast cancer cells an early advantage in tumor initiation. The cause of this early advantage could be linked to decreased apoptosis of the cells through decreased expression of the pro-apoptotic ligand TRAIL [51,52]. Mice injected with 468-ΔTC cells were also found to have increased metastatic burden to the lymph nodes indicating increased severity of metastasis. The increased activity of the uPA system could help the tumor metastasize more readily. In addition, the decreased adhesion and expression of MCAM in 468-ΔTC cells could also help to explain why there is an increased effect on lymph node metastasis.

The bulk of evidence concerning OPN function implicates the integrin binding domains in OPN-mediated tumorigenesis [14-18,20-24]. However, some studies show that it is the C-terminal half of OPN, containing the CD44 binding domain, that is responsible for the protein's effects on cancer cells [31,53-55]. We hypothesize that deletion of the thrombin cleavage domain may be interrupting integrin binding of OPN and therefore it could be CD44 binding to OPN which may be causing the observed effects. Future studies to test this hypothesis are currently being investigated.

## Conclusions

In summary, the novel findings presented here demonstrate that deletion of the thrombin cleavage domain from OPN decreases *in vitro *cell adhesion and *in vivo *primary tumor latency time, and increases *in vitro *uPA expression and activity as well as *in vivo *primary tumor growth and lymph node metastatic burden of MDA-MB-468 breast cancer cells. To the best of our knowledge, this is the first study to specifically study the effects of OPN thrombin cleavage by site-directed mutagenesis. Taken together, the results presented here suggest that thrombin-uncleavable OPN conveys an early tumor formation advantage as well as a metastatic advantage, possibly due to increases in proteolytic activity and decreases in adhesion and apoptosis. Further studies are needed to clarify whether ΔTC-OPN is simply refractory to cleavage, or whether the deletion imparts some other additional effects such as altering the conformation of OPN and/or inhibiting an undefined interaction that in turn influence downstream functional cell behavior. Elucidation of the mechanisms responsible for these observations and the translation of this knowledge into the clinic could ultimately provide new therapeutic opportunities for combating breast cancer.

## Abbreviations

468-CON: MDA-MB-468 cells stably transfected with a control vector; 468-OPN: MDA-MB-468 cells stably transfected with wildtype osteopontin; 468-ΔTC: MDA-MB-468 cells stably transfected with osteopontin containing a deleted thrombin cleave domain; αMEM: alpha minimum essential medium; ANOVA: analysis of variance; cDNA: complimentary deoxyribonucleic acid; DTT: dithiothreitol; EGFR: epidermal growth factor receptor; FBS: fetal bovine serum; GAPDH: glyceraldehyde 3-phosphate dehydrogenase; GLRSKS: glycine-leucine-arginine-serine-lysine-serine; H&E: hematoxylin and eosin; HPF: high-powered field; HRP: horseradish peroxidase; IgG: immunoglobulin G; LN: lymph node; MCAM: melanoma cell adhesion molecule; OPN: osteopontin; PBS: phosphate buffered saline; PCR: polymerase chain reaction; PE: phycoerytherin; RGB: red green blue; RGD: arginine-glycine-aspartic acid; RNA: ribonucleic acid; RSK: arginine-serine-lysine; SEM: standard error of the mean; SERPINB5: serine protease inhibitor B5; SDS: sodium dodecyl sulfate; SVVYLR: serine-valine-valine-tyrosine-leucine-arginine; TBST: tris-buffered saline tween; TF: tissue factor; TRAIL: TNF-related apoptosis-inducing ligand; uPA: urokinase plasminogen activator; VN: vitronectin.

## Competing interests

The authors declare that they have no competing interests.

## Authors' contributions

MSB participated in the design of the study, carried out the majority of the experimental work, and drafted the manuscript. EBS contributed to the western blot analysis and the adhesion assays. DG performed the tumor cell injections for the *in vivo *mouse assays. COP carried out the histopathological sectioning and staining of mouse tissues. ALA conceived of the study, participated in its design and coordination, and helped to draft the manuscript. All authors read and approved the final manuscript.

## Pre-publication history

The pre-publication history for this paper can be accessed here:

http://www.biomedcentral.com/1471-2407/11/25/prepub

## Supplementary Material

Additional file 1 **Figure S1. Expression of various cell surface integrin receptors and CD44 receptor in the 468-CON, 468-OPN, and 468-ΔTC cell lines**. Expression was measured by flow cytometry analysis as described in the Materials and Methods. Cultured cells were incubated with specific antibodies (*filled profiles*) or with a nonspecific isotype control primary antibody (*open profiles*). Filled profiles represent expression of α9β1 integrin, β1 integrin, β3 integrin, αvβ5 integrin and CD44. Click here for file

Additional file 2 **Figure S2. *In vitro *cell proliferation and migration of 468-CON, 468-OPN and 468-ΔTC cells**. ***(A) ***Cell growth kinetics in normal culture over time of 468-CON (*black squares*), 468-OPN (*open circles*) and 468-ΔTC cells (*open triangles*) (n = 3 plates/timepoint). Data are presented as the mean ± SEM. ***(B) ***Cell migration of 468-CON (*black bars*), 468-OPN (*white bars*), and 468-ΔTC cells (*hatched bars*) towards 10% fetal bovine serum (FBS). Transwells (8 μm) were pre-coated with gelatin (6 μg/well) and cells (5 × 10^4 ^cells/well; n = 3 for each treatment) were allowed to migrate for 24 hrs. Migrated cells were quantified by manual counting of 5 HPF per well. Data are presented as the mean ± SEM. * = significantly different than 468-CON cells (p < 0.05). In all cases, data are compiled from at least three separate experiments. Click here for file
