# Supplementary figures and images for: Deletion of the thrombin cleavage domain of osteopontin mediates breast cancer cell adhesion, proteolytic activity, tumorgenicity, and metastasis
Source: BMC Cancer. 2011 Jan 19;11:25. doi: 10.1186/1471-2407-11-25 (PMC3034707; doi:10.1186/1471-2407-11-25)

SUPPLEMENTAL FIGURE 1- Beausoleil et al 2010

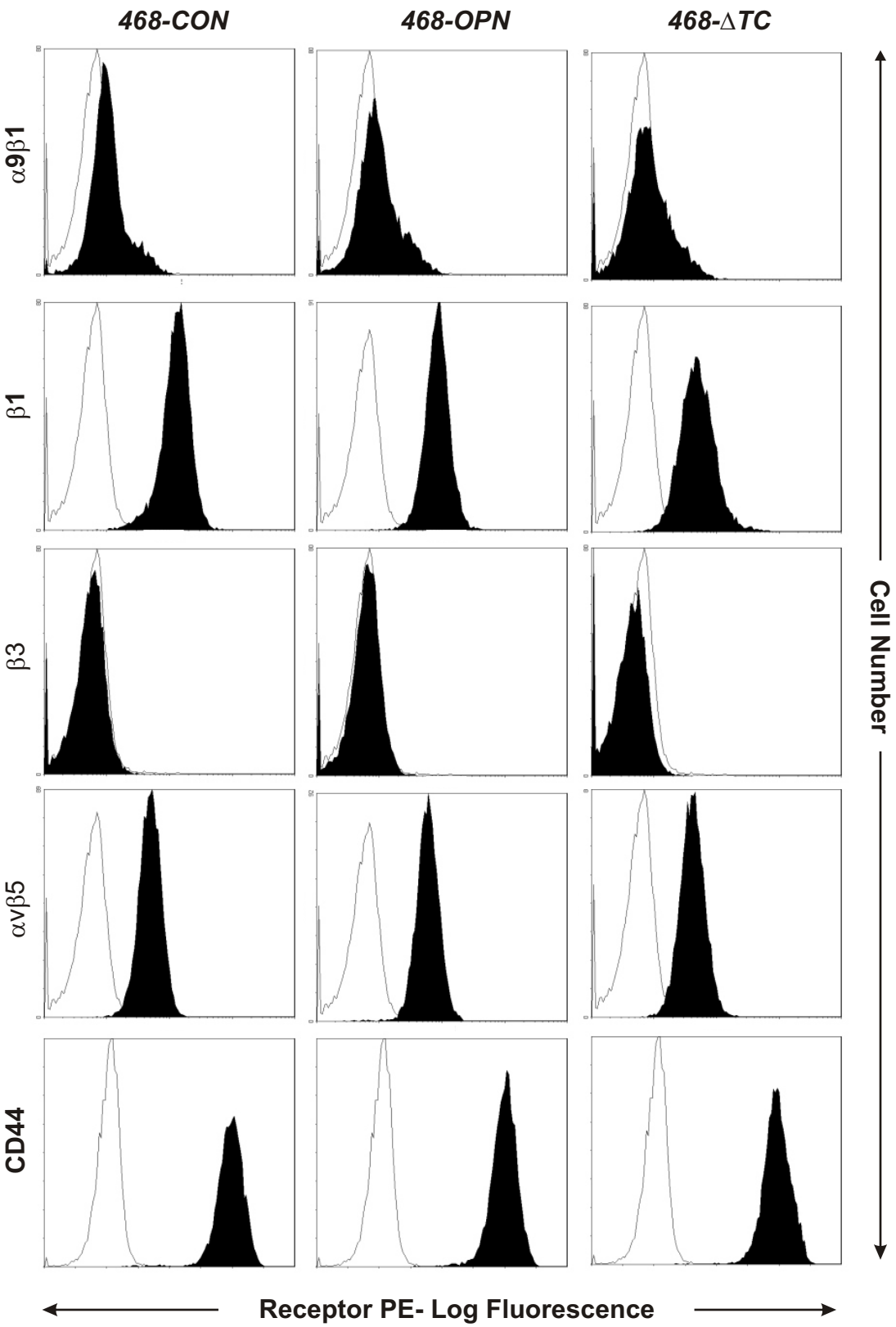

Supplement: Additional file 1 — Figure S1. Expression of various cell surface integrin receptors and CD44 receptor in the 468-CON, 468-OPN, and 468-ΔTC cell lines. Expression was measured by flow cytometry analysis as described in the Materials and Methods. Cultured cells were incubated with specific antibodies (filled profiles) or with a nonspecific isotype control primary antibody (open profiles). Filled profiles represent expression of α9β1 integrin, β1 integrin, β3 integrin, αvβ5 integrin and CD44. [file 1471-2407-11-25-S1.PDF]

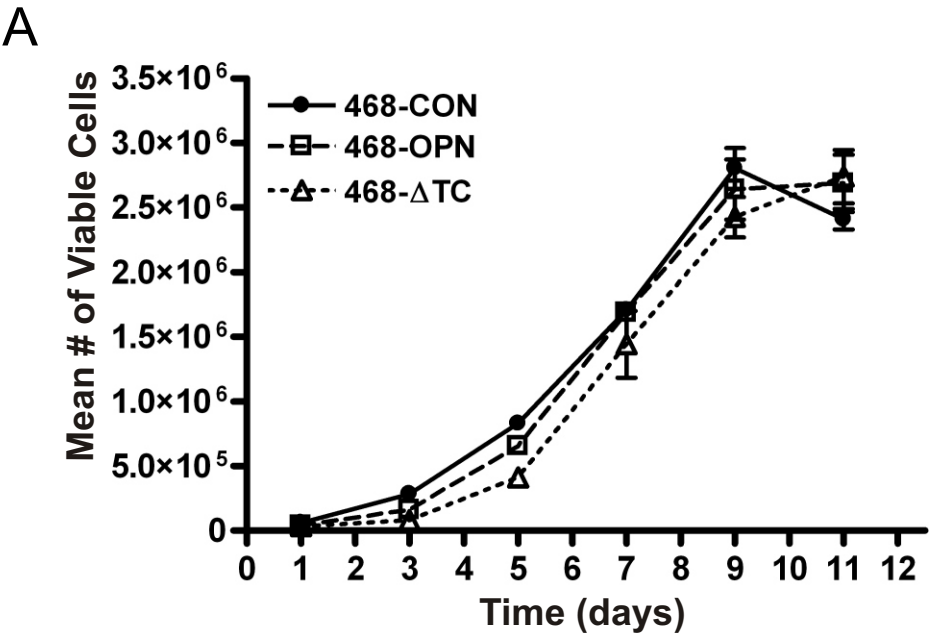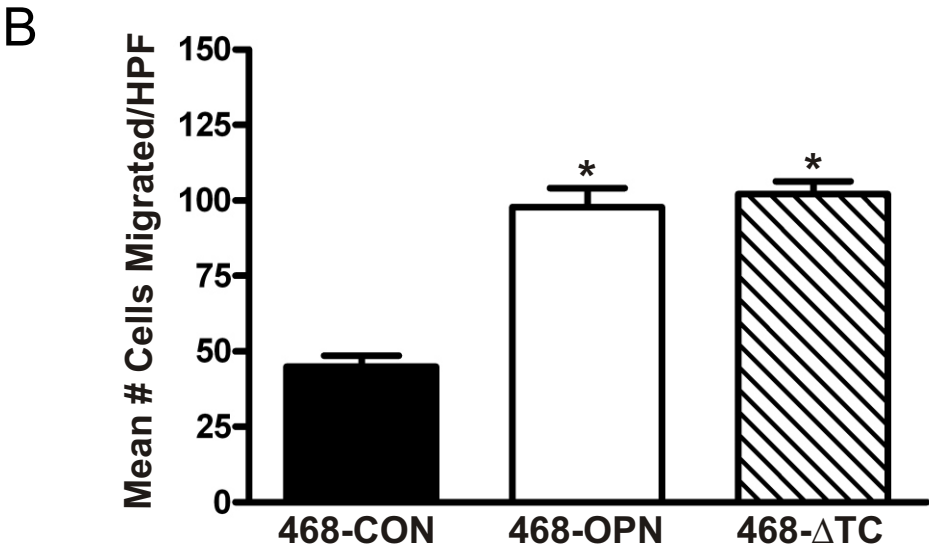

Supplement: Additional file 2 — Figure S2. In vitro cell proliferation and migration of 468-CON, 468-OPN and 468-ΔTC cells. (A) Cell growth kinetics in normal culture over time of 468-CON (black squares), 468-OPN (open circles) and 468-ΔTC cells (open triangles) (n = 3 plates/timepoint). Data are presented as the mean ± SEM. (B) Cell migration of 468-CON (black bars), 468-OPN (white bars), and 468-ΔTC cells (hatched bars) towards 10% fetal bovine serum (FBS). Transwells (8 μm) were pre-coated with gelatin (6 μg/well) and cells (5 × 10^4^ cells/well; n = 3 for each treatment) were allowed to migrate for 24 hrs. Migrated cells were quantified by manual counting of 5 HPF per well. Data are presented as the mean ± SEM. * = significantly different than 468-CON cells (p < 0.05). In all cases, data are compiled from at least three separate experiments. [file 1471-2407-11-25-S2.PDF]
